# Multi-country surveillance of paediatric invasive group A *Streptococcus* infection, European Union/European Economic Area countries, 2022/23 season

**DOI:** 10.2807/1560-7917.ES.2025.30.42.2500079

**Published:** 2025-10-23

**Authors:** Maria João Cardoso, Dorothée Obach, Emma Löf, Gaetano Marrone, Laura Cornelissen, Myrofora Charalambous, Sandra Vohrnova, Celine Plainvert, Asmaa Tazi, Theano Georgakopoulou, Cilian Ó Maoldomhnaigh, Orla Cotter, Paul McKeown, Brechje de Gier, Barbro Mäkitalo, Agoritsa Baka, Vivian H Leung

**Affiliations:** 1 Institute of Medical Microbiology and Hygiene, Austrian Agency for Health and Food Safety, Vienna, Austria; 2 ECDC Fellowship Programme, Public Health Microbiology path (EUPHEM), European Centre for Disease Prevention and Control (ECDC), Stockholm, Sweden; 3 European Centre for Disease Prevention and Control (ECDC), Stockholm, Sweden; 4 Epidemiology and Public Health, Sciensano, Brussels, Belgium; 5 Archbishop Makarios III Hospital, State Health Services Organisation, Cyprus; 6 National Reference Laboratory for Streptococcal Infections, Department of Air-Borne Bacterial Infections, Centre for Epidemiology and Microbiology, National Institute of Public Health, Prague, Czechia; 7 Third Faculty of Medicine, Charles University, Prague, Czechia; 8 Department of Bacteriology, National Reference Centre for Streptococci, University Hospital Federation Perinatal Inflammation and Prematurity, Assistance Publique – Hôpitaux de Paris, Paris Centre, Hôpital Cochin, Paris, France; 9 Team «Bacterial Pathogenesis and Innate Immune Signalling», Université Paris Cité, Institut Cochin, Inserm U1016, CNRS UMR8104, Paris, France; 10 Department for Vaccine Preventable Diseases and Congenital Infections, National Public Health Organization, Athens, Greece; 11 Paediatric Infectious Disease Department, Children’s Health Ireland, Dublin, Ireland; 12 HSE Department of Public Health Dublin Midlands, Health Service Executive, Dublin, Ireland; 13 National Health Protection Office, Health Service Executive, Dublin, Ireland; 14 Center for Infectious Disease Control, National Institute for Public Health and the Environment (RIVM), Bilthoven, the Netherlands; 15 Public Health Agency of Sweden, Solna, Sweden

**Keywords:** invasive disease, Streptococcus pyogenes, case-case study, Europe, international collaboration

## Abstract

**BACKGROUND:**

Group A *Streptococcus* (GAS) commonly causes mild bacterial infections but also deadly invasive disease. An upsurge in paediatric invasive GAS (iGAS) infections was observed during the last quarter of 2022 in the European Union/European Economic Area (EU/EEA) countries.

**AIM:**

We aimed to assess iGAS surveillance in the EU/EEA countries and investigate the epidemiology of iGAS infections during the 2022/23 season.

**METHODS:**

We conducted a study on GAS and iGAS surveillance to evaluate coverage and surveillance methodology across the EU/EEA countries. We collected and analysed data on paediatric iGAS cases (patients aged ≤ 16 years) occurring in September 2022–June 2023 that resulted in hospitalisation or death. Associations of severe outcome (admission to intensive care unit and/or death) with potential risk factors were estimated by logistic regression in a case-case analysis.

**RESULTS:**

Nineteen countries responded to the questionnaire; eleven had mandated national surveillance for iGAS before 2022. Eight countries submitted data on 1,277 paediatric iGAS cases involving hospitalisation or death: 56% were males and median age was 4 years. Sixty-three (5%) of these cases died. Severe outcome was associated with *emm*1 type (odds ratio (OR) = 1.73; 95% confidence interval (CI): 1.13–2.67), having a sepsis without a known anatomic source (OR = 1.73; 95% CI: 1.11–2.73) and lower respiratory tract infections (OR = 4.14; 95% CI: 2.70–6.44).

**CONCLUSION:**

Surveillance of GAS and iGAS infections varied among the participating countries. We highlight the importance of including *emm* typing and analysis of clinical data in iGAS surveillance and having international collaboration for effective response to future surges.

**Key public health message**

**What did you want to address in this study and why?**

Group A *Streptococcus* (GAS) bacteria can cause severe disease, known as invasive GAS or iGAS. We aimed to assess surveillance of iGAS in European countries and factors associated with severe illness and death among children and adolescents with iGAS.

**What have we learnt from this study?**

The level of surveillance for iGAS infections varied among the countries. A harmonised case definition facilitated pooling of data from 1,277 cases across eight countries for this study. We found young age, female sex, sepsis, lower respiratory tract infection, and infection with *emm*1 strain to be associated with higher risk of admission to an intensive care unit and/or death.

**What are the implications of your findings for public health?**

This study highlights the value of multi-country collaborations in strengthening public health responses to infections not routinely under surveillance. Harmonised case definitions, monitoring of *emm* types and antimicrobial susceptibility, integrated surveillance with viral respiratory pathogens and collaborative frameworks are key to improving early warning, cross-border preparedness and response to emerging infections.

## Introduction

Group A *Streptococcus* (GAS), also known as *Streptococcus pyogenes*, is a pathogen causing a broad spectrum of disease in humans, from mild non-invasive (e.g. scarlet fever, pharyngitis and impetigo) to life-threatening invasive infections such as bacteraemia or sepsis, necrotising fasciitis, streptococcal toxic shock syndrome (STSS) and pneumonia [1]. Invasive GAS (iGAS) infections can cause considerable morbidity and mortality, often requiring hospitalisation, with a case-fatality ranging from 3.6% to 8.3% in children [2]. Risk factors for rapid progression of invasive disease include young or advanced age, pregnancy and chronic conditions such as immunodeficiency, viral co-infection or recent viral infection [3,4]. Despite iGAS being an important cause of morbidity and mortality, no vaccines are currently available [5].

The rapid and efficient transmission of this bacterium within certain populations can lead to outbreaks of invasive infections, creating a substantial public health burden [6]. The risk of iGAS infection is increased in households with a recent case of iGAS or scarlet fever, and pharyngeal GAS carriage among close contacts [7,8], which supports the hypothesis of person-to-person transmission of GAS by respiratory droplet or skin contact [9,10]. Outbreaks of GAS are not fully explained by direct contact alone, suggesting a role for indirect contact and airborne transmission, particularly in school and childcare settings [11]. In addition, widespread GAS outbreaks can be associated with diverse expression of virulence factors or mutations in the bacteria. The M protein is a major virulence factor for GAS, and the gene encoding it (*emm* gene) is used for genotyping. Increases in GAS and iGAS cases in England in the 2010s were associated with emergence of the hypervirulent M1_UK_ clone which in 2013 outnumbered the M1_global_ strain circulating since the 1980s [12,13].

During the last quarter of 2022, surges in iGAS cases were reported by several countries including France, Ireland, the Netherlands, Spain and the United Kingdom (UK), particularly among paediatric populations [14-16] but also among adults [17]. In 2023, a European Union (EU) country requested the EU Health Task Force (EUHTF) to coordinate further investigation of the unusually high paediatric morbidity and mortality associated with iGAS. The EUHTF is an initiative led by European Centre for Disease Prevention and Control (ECDC), established in 2023 to support emergency preparedness and crisis response, including operational research. For this study, the EUHTF gathered a team of experts to facilitate an investigation of paediatric iGAS cases across multiple countries during the 2022/23 season. The aim of the investigation was to assess iGAS surveillance in the EU/European Economic Area (EU/EEA) countries and identify risk factors potentially associated with increased likelihood of severe disease and death from iGAS.

Paediatric iGAS disease is uncommon, so collecting and pooling of harmonised data at an EU level could potentially yield more valid conclusions than single-country studies. Thus, ECDC invited EU/EEA countries to participate in a retrospective study with the following objectives: (i) evaluate the availability and quality of GAS or iGAS surveillance data; (ii) describe paediatric iGAS cases during the 2022/23 season; and (iii) assess available data on paediatric iGAS cases in participating countries to better understand potential factors associated with severe illness and death, with the goal of supporting public health actions.

## Methods

### Questionnaire on surveillance systems for invasive and non-invasive group A *Streptococcus* infections

To assess coverage and methodologies of existing surveillance systems for GAS and iGAS infections, ECDC National Focal Points for Surveillance from all 30 EU/EEA countries were invited to respond to a questionnaire about GAS and iGAS surveillance in their country. The questionnaire, presented in Supplementary File 1, was administered via REDCap (https://project-redcap.org) between 21 September and 3 October 2023.

We used the responses to describe existing iGAS surveillance before 2022 and changes to surveillance or new surveillance methods established in 2022 or 2023. The responses also revealed whether GAS surveillance was used to capture iGAS cases, whether laboratories and/or clinicians notified cases to public health authorities, whether molecular typing was done during the 2022/23 season, and types of case data available to the national public health institutes.

### Data from paediatric cases of invasive group A *Streptococcus* infection

All EU/EEA countries with paediatric iGAS case data available were invited to contribute to a multi-country study via nominated national contact points for iGAS. We generated a case report form (CRF) for these data using REDCap hosted at ECDC. The form was also available in Microsoft Excel upon request. Countries contributed from 1 August 2023 to 31 January 2024 by sharing de-identified case data after signing a data sharing agreement.

Case definitions are described in Box.

Box **Study definition of paediatric case with invasive group A *Streptococcus* (iGAS) infection, the European Union/European Economic Area, 1 September 2022–30 June 2023**

**Paediatric case:**

• Illness in a person aged ≤ 16 years resulting in hospitalisation or death from 1 September 2022 to 30 June 2023

AND

• Detection of group A *Streptococcus*

o By culture or by PCR from a specimen from a normally sterile body site (blood, cerebral spinal fluid, pericardium, peritoneum, pleural fluid, endometrium, joint aspirate, bone, deep tissue) or from a deep abscess at operation or at post-mortem

OR

o By culture or by immunoassay (rapid streptococcal test) or PCR from a specimen from a non-sterile body site (e.g. throat, ear, sputum, skin and nails, vagina, penis, anus, open wounds) AND

o Clinical presentation consistent with severe streptococcal infection (STSS, puerperal sepsis, necrotising fasciitis, myositis, pneumonia, septic arthritis, meningitis, peritonitis, osteomyelitis, cellulitis with systemic presentation).

STSS: streptococcal toxic shock syndrome.

The CRF included 109 data fields divided into seven sections: patient demographics and dates; healthcare visits before admission; clinical presentation at hospital admission; viral co-infections and medical predisposition; GAS microbiological testing; course of illness and treatment; outcomes, as presented in Supplementary File 2. National contact points extracted these data from epidemiological databases and, when possible, medical records.

### Data analysis

Case-case studies compare cases of a given disease with a specific characteristic with other cases with the same disease but without the characteristic. In this study, we conducted case-case analyses comparing groups of cases with different severities. We classified cases that met the case definition into three groups: (i) cases admitted to a hospital ward; or (ii) to an intensive care unit (ICU); and (iii) cases who died. These were used as proxies for the degree of disease severity and treated as mutually exclusive, meaning each case was classified to the group representing the highest level of severity observed. Reporting of some variables was required to validate case classification, such as date of data extraction, admission to a hospital ward and/or an ICU, date of illness onset, age and death status. We excluded cases lacking both ward and ICU admission status (unable to assign group) from the case-case analyses but included deaths with unknown history of hospital admission. The Netherlands reported 101 hospitalised cases but could not determine ICU status; these cases were thus excluded from the case-case analyses. For all other cases, missing values for the variables ICU admission and death were assumed to be negative (e.g. no ICU admission, no death).

We grouped the cases into four age groups (0–2 years, 3–5 years, 6–10 years and 11–16 years) to reflect differences in physiological development and social interaction, considering preschool and school groupings, and also the potential for the youngest age group to be GAS-naïve due to reduced exposure during the COVID-19 pandemic.

We created two composite variables ‘previous viral infection’ and ‘underlying conditions’ from the data. In the previous viral infections, we included infections in the previous 30 days due to influenza virus, respiratory syncytial virus (RSV), severe acute respiratory syndrome coronavirus 2 (SARS-CoV-2), other respiratory viruses and varicella zoster virus. Underlying conditions considered were asthma, other chronic respiratory disease, diabetes and other immunocompromising conditions. The two composite variables were used as independent variables in the analysis.

We grouped diagnoses associated with iGAS into seven groups, as presented in Supplementary Table S1. We created a ‘sepsis only’ group to distinguish sepsis cases without a known anatomic source from those with additional diagnoses. Cases with multiple diagnoses were included in multiple diagnosis groups. We grouped reported GAS *emm* types into a dichotomous variable, distinguishing between *emm*1 and non-*emm*1 types.

#### Statistical methods

We carried out data management and statistical analysis using R version 4.3.1 (June 2023) (https://www.r-project.org/), REDCap 14.1.4 and Microsoft Excel. We conducted bivariate analyses of factors associated with ICU admission, death and severe outcome (defined as ICU admission and/or death) using chi-square or Fischer’s exact test.

We included all factors significantly associated with severe outcome in the bivariate analysis (p < 0.10) in multivariable models to calculate odds ratios (OR) with 95% confidence intervals (CI). Two multivariable logistic regression models were fitted using the glm() function in R and built following the principle of parsimony. The first multivariable model, for analysis of case characteristics associated with severe outcome, included all cases for which severity (ward admission, ICU admission and/or death) and all independent variables could be determined from reported data. The second multivariable model, for severity analysis of cases with typed isolates, *emm* type was added as an independent variable together with severity and the other independent variables from the first model. Cases with missing values for variables studied in each model were excluded from that respective model. Cardiac group could not be included in the multivariable models because there were too few cases.

## Results

### Participating countries

**Figure 1:**
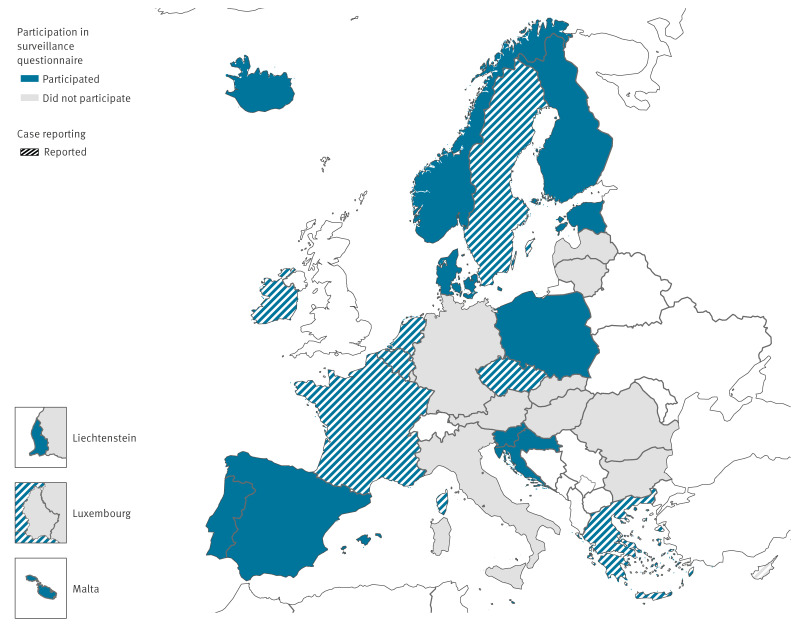
Map of countries participating in a survey on surveillance of non-invasive and invasive infections with group A *Streptococcus* (GAS) and data on paediatric cases with invasive GAS (iGAS) infections, European Union/European Economic Area countries, 2023 (n = 19)^a^

Nineteen EU/EEA countries (Belgium, Croatia, Czechia, Denmark, Estonia, Finland, France, Greece, Iceland, Ireland, Liechtenstein, Malta, the Netherlands, Norway, Poland, Portugal, Slovenia, Spain, Sweden) responded to the questionnaire about public health surveillance of non-invasive GAS and iGAS, and eight countries (Belgium, Cyprus, Czechia, France, Greece, Ireland, the Netherlands, Sweden) submitted paediatric iGAS case data (Figure 1).

ECDC: European Centre for Disease Prevention and Control.

^a^ Paediatric iGAS case: illness in a person aged 0–16 years characterised by detection of group A *Streptococcus* from a normally sterile body site, or from a non-sterile body site in combination with a clinical presentation consistent with severe streptococcal infection, resulting in hospitalisation or death from 1 September 2022 to 30 June 2023.

Administrative boundaries: EuroGeographics. The boundaries and names shown on this map do not imply official endorsement or acceptance by the European Union. Map produced by ECDC on 5 July 2024.

### Surveillance of non-invasive and invasive group A *Streptococcus* infections

**Figure 2:**
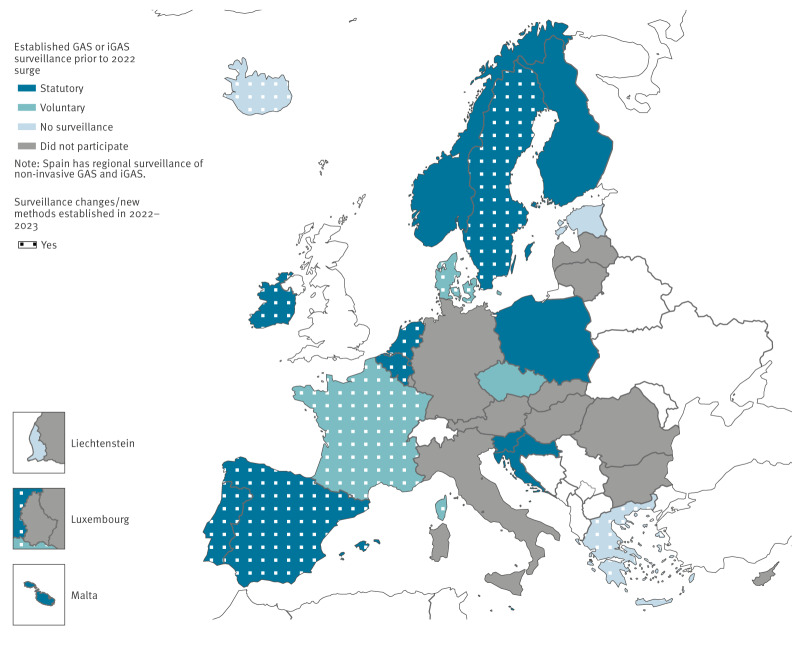
Surveillance of group A *Streptococcus* (GAS) and/or invasive GAS (iGAS) infections before 2022 and changes implemented 2022–2023, European Union/European Economic Area countries (n = 19)^a^

According to the questionnaire responses, before 2022, surveillance of GAS and iGAS infections varied (Figure 2). Croatia, Poland and Slovenia had statutory national reporting of GAS and iGAS cases. Spain had only statutory regional surveillance of GAS and iGAS. Eight countries had mandatory notification of iGAS infections only (Belgium, Finland, Ireland, Malta, the Netherlands, Norway, Portugal and Sweden). Seven countries had no mandatory notification (Czechia, Denmark, Estonia, France, Greece, Iceland and Liechtenstein), three of which had voluntary sharing of case information and isolates with the national public health institutes (Czechia, Denmark, France).

ECDC: European Centre for Disease Prevention and Control.

^a^ Reported establishment of any surveillance system for invasive or non-invasive Group A *Streptococcus* infections are indicated. Case definitions varied between countries.

Administrative boundaries: EuroGeographics. The boundaries and names shown on this map do not imply official endorsement or acceptance by the European Union. Map produced by ECDC on 5 July 2024.

Ten countries adopted new or supplementary iGAS surveillance methods in 2022–2023: four countries implemented new retrospective surveillance methods, four countries implemented new prospective methods, and two countries implemented both. For case finding, ten countries had both laboratory and clinician-based case notification, four countries relied only on laboratories, and two countries relied only on clinicians. There was also heterogeneity in laboratory-based case finding methods: six countries included all laboratory findings of GAS, five countries only included isolates from sterile sites, and three countries included isolates from non-sterile sites if there was systemic disease.

Eight countries did not routinely type iGAS case isolates and three countries did not respond about routine typing. Among the eight that did, approaches to typing varied: two countries routinely used whole genome sequencing (WGS) and six countries conducted routine *emm* typing using other molecular techniques.

Ten countries indicated that medical chart reviews would be necessary for the public health institute to obtain all the basic epidemiological data required for our study, as information on ICU admission status and date, symptom onset, type of infection and diagnosis information at admission/discharge was often missing in case reporting. Nine countries indicated availability of clinical laboratory test results at the public health institute for viruses including influenza virus, varicella zoster virus and RSV.

### Data on paediatric cases with invasive group A *Streptococcus* infections

We received data from 1,277 paediatric cases of iGAS infections that resulted in hospitalisation or death from eight EU countries. The completeness of the data varied considerably among the variables studied, with high proportions for age and sex (100% and 99.8%, respectively) and low proportions for previous viral illness (n = 280; 22%) and underlying conditions (n = 244; 19%). At least one clinical diagnosis was reported for 1,244 (97%) cases, and data on *emm* typing were available for 689 (54%) cases.

The highest case-fatality was reported by Greece (19%), followed by Czechia (9%), and the highest percentage of ICU admission was reported by Cyprus (50%), followed by Czechia (31%) and Belgium (21%) (Table 1). Nineteen (30%) of the 63 paediatric iGAS deaths occurred without record of hospitalisation.

**Table 1:** Paediatric cases with invasive group A *Streptococcus* infections, by country, eight European Union countries, 1 September 2022–30 June 2023 (n = 1,277)

Country	Ward admitted (n = 904)		ICU admitted (n = 209)^a^		Deaths (n = 63)^a^		Unable to assign group (n = 101)	Total	%
	n	%	n	%	n	%			
Belgium	158	74	46	21	10	5	0	214	17
Cyprus	3	50	3	50	0	0	0	6	0.5
Czechia	39	60	20	31	6	9	0	65	5
France	430	76	107	19	26	5	0	563	44
Greece	24	75	2	6	6	19	0	32	2.5
Ireland	126	81	24	15	5	3	0	155	12
The Netherlands	Not available				4	4	101	105	8
Sweden	124	91	7	5	6	4	0	137	11

ICU: intensive care unit.

^a^ Cases with missing data on admission to ICU or death but information on ward admission were included in the ward group.

**Figure 3:**
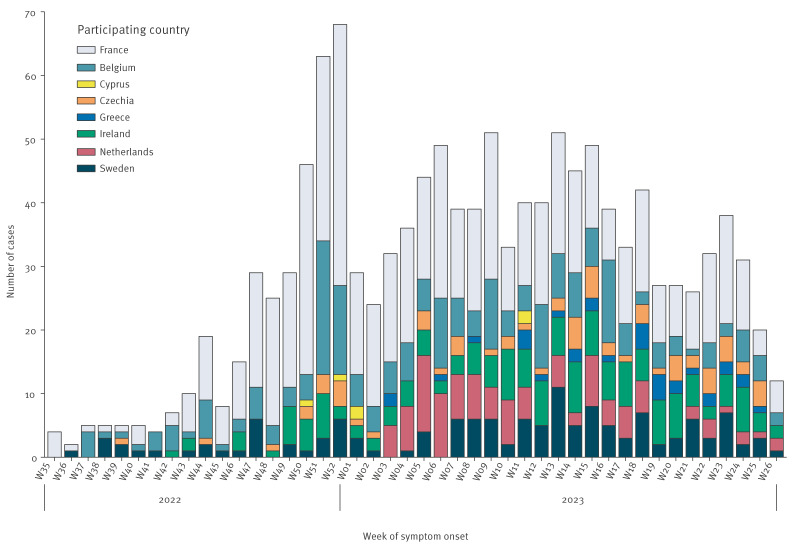
Epidemic curve of the distribution of paediatric cases with invasive group A *Streptococcus* infections, by week of symptom onset, European Union/European Economic Area countries, 1 September 2022–30 June 2023 (n = 1,277)

Of the 1,277 cases, 711 (56%) were males and 564 (44%) were females (two unknown sex); the median age was 4 years (interquartile range (IQR): 1–7 years). The age group of 0–2 years had most cases (n = 470; 37%) and the age group of 11–16 years fewest (n = 122; 10%). Case numbers decreased with increasing age (Table 2). Case numbers by age are also presented in Supplementary Figure S1. The epidemic curve shows a peak in case numbers at the end of 2022, followed by a sustained wave during the first quarter of 2023 (Figure 3).

**Table 2:** Characteristics of paediatric cases with invasive group A *Streptococcus* infections, eight European Union countries, 1 September 2022–30 June 2023 (n = 1,277)^a^

Characteristic	Ward admitted (n = 904)		ICU admitted (n = 209)^b^		Deaths (n = 63)^b^		Unable to assign group (n = 101)	Total	%
	n	%	n	%	n	%			
Sex^b^									
Female	370	41	109	52	36	57	49	564	44
Male	532	59	100	48	27	43	52	711	56
Unknown	2	0	0	0	0	0	0	2	0.2
Age group (years)^b^									
0–2	320	35	95	45	23	37	32	470	37
3–5	273	30	51	24	24	38	32	380	30
6–10	227	25	41	20	9	14	28	305	24
11–16	84	9	22	11	7	11	9	122	10
Previous viral illness^b^									
Yes	171	17	47	22	9	14	0	227	18
No	45	5	7	3	1	2	0	53	4
Unknown	688	79	155	74	53	84	101	997	78
Underlying conditions^b^									
Yes	24	3	9	4	1	2	0	34	3
No	167	18	33	16	10	16	0	210	16
Unknown	713	79	167	80	52	83	101	1,033	81
Diagnosis^b^									
All sepsis diagnoses^c^	352	NA	99	NA	44	NA	37	532	42
Sepsis only	258	29	59	28	34	54	30	381	30
Lower respiratory tract diagnoses^c^	132	NA	87	NA	17	NA	10	246	19
Skin and soft tissue diagnoses^c^	188	NA	33	NA	2	NA	9	232	18
Musculoskeletal diagnoses^c^	127	NA	8	NA	1	NA	8	144	11
Upper respiratory tract diagnoses^c^	49	NA	4	NA	1	NA	4	58	5
Cardiac diagnoses^c^	0	NA	0	NA	2	NA	0	2	0.2
Other^c^	31	NA	8	NA	1	NA	10	50	4

ICU: intensive care unit; NA: not applicable.

^a^ The countries included are Belgium, Cyprus, Czechia, France, Greece, Ireland, the Netherlands, Sweden.

^b^ Cases with missing data on admission to ICU or death but information on ward admission were included in the ward group.

^c^ A case could have more than one of these diagnoses.

W: week.

After removing the 101 hospitalised cases from the Netherlands for which ICU admission status could not be determined, 1,176 cases from eight countries were assigned to a severity group. The sex and age distributions of the 1,176 cases analysed were similar to those for all 1,277 reported cases (males: n = 659; 56%; females: n = 515; 44%; median age: 4 years (IQR: 1–7 years)). Of the 1,176 cases, 77% (n = 904) were admitted to a non-ICU hospital ward, 18% (n = 209) to an ICU, and 5% (n = 63) died. Of the cases admitted to an ICU, 100 (48%) were males, 109 (52%) were females, and the median age was 3 years (IQR: 1–6 years). Twenty-seven (43%) of the cases who died were males, 36 (57%) were females, and the median age was 3 years (IQR: 1–5.5 years). Almost 70% (n = 146) of the cases admitted to the ICU, and 75% (n = 47) of those who died were aged ≤ 5 years.

Among the 532 cases with a diagnosis of sepsis, 151 (28%) had a second diagnosis, and 381 (72%) had ‘sepsis only’. The lower respiratory tract was the most reported site of disease in iGAS cases (n = 246): 220 (89%) of the cases with lower respiratory tract diagnoses had a diagnosis of pneumonia, 26 (11%) had empyema and 18 (7%) had both empyema and pneumonia.

Information on *emm* type was submitted from 689 cases from Czechia, France, Greece and Sweden (Table 3). These cases had similar characteristics as the other cases: median age of 4 years, 400 (58%) were males, 289 (42%) females, 516 (75%) admitted to a ward, 131 (19%) ICU and 42 (6%) died. Czechia was the only country with *emm* typing results for all reported cases. Typing results were available for 561 of 563 cases from France, 22 of 32 cases from Greece and 42 of 137 cases from Sweden. The most common *emm* type was *emm*1, found in 422 (61%) isolates, followed by *emm*12 (n = 141; 20%).

**Table 3:** Results on *emm* types of group A *Streptococcus* isolates from paediatric cases, Czechia, France, Greece and Sweden, 1 September 2022–30 June 2023 (n = 689)

M type	Country				Total	%
	Czechia	France	Greece	Sweden		
*emm*1	40	356	10	16	422	61
*emm*12	15	104	10	12	141	21
*emm*89	2	23	0	0	25	4
*emm*4	1	14	1	5	21	3
*emm*75	2	13	0	0	15	2
*emm*87	1	13	0	1	15	2
*emm*77	0	7	0	2	9	1
*emm*28	1	5	0	1	7	1
Other *emm* types^a^	3	26	1	4	34	5
Total	65	561	22	41	689	100

^a^ Other *emm* types include all types with ≤ 5 findings.

### Case-case analysis of paediatric cases with invasive group A *Streptococcus* infections

Sex, age groups, ‘sepsis only’, lower respiratory tract diagnoses, upper respiratory tract diagnoses, skin and soft tissue diagnoses, and musculoskeletal diagnoses were found to be associated with severe outcome (p < 0.10) in bivariate analyses, and these variables were included in the multivariable models (Table 4). Cardiac diagnoses and all sepsis diagnoses were also found to be associated with severe outcome in bivariate analyses; however, these two variables were not included in multivariable models. Too few cases had cardiac diagnoses for the multivariable modelling, and sepsis diagnoses were excluded because these cases had other diagnoses included in the models.

**Table 4:** Bivariate and multivariable analysis of severe outcome of paediatric cases with invasive group A *Streptococcus* infection, eight European Union countries, 1 September 2022–30 June 2023 (n = 1,176)^a, b^

Variable	Bivariate analysis (n = 1,176)^c^				Case-case severity analysis (n = 1,174)			Severity analysis for cases with typed isolates (n = 689)		
	OR	95% CI		p value	OR	95% CI	p value	OR	95% CI	p value
Sex										
Female	Reference				Reference			Reference		
Male	0.609		0.46–0.80	< 0.001	0.57	0.43–0.76	< 0.001	0.62	0.42–0.92	0.018
Age (years)										
0–2	Reference p value: 0.039				Reference			Reference		
3–5	0.75		0.56–0.98	0.081	0.72	0.50–1.02	0.063	0.90	0.56–1.46	0.700
6–10	0.6		0.43–0.82	0.006	0.62	0.42–0.92	0.018	0.79	0.46–1.34	0.400
11–16	0.94		0.63–1.38	0.793	1.01	0.61–1.65	0.900	1.33	0.69–2.50	0.400
Clinical condition										
Sepsis only	1.3		1.02–1.66	0.074	1.73	1.11–2.73	0.016	0.97	0.53–1.80	0.900
Lower respiratory tract diagnoses	3.62		2.65–4.91	< 0.001	4.14	2.70–6.44	< 0.001	3.66	2.05–6.62	< 0.001
Upper respiratory tract diagnoses	0.34		0.11–0.77	0.026	0.37	0.17–0.72	0.006	0.23	0.05–0.71	0.029
Skin and soft tissue diagnoses	0.57		0.37–0.82	0.003	0.92	0.56–1.50	0.700	0.40	0.21–0.75	0.005
Musculoskeletal diagnoses	0.21		0.10–0.40	< 0.001	0.50	0.26–0.90	0.025	0.15	0.05–0.38	< 0.001
All sepsis diagnoses	1.74		1.32–2.28	< 0.001	NA					
Cardiac diagnoses	Inf		0.63–Inf	0.053						
Other diagnoses	0.96		0.40–2.11	0.923						
Previous viral infection	1.81		0.84–4.39	0.163						
Underlying conditions	1.63		0.69–3.60	0.287						
M type										
*emm*1 type	2.06		1.42–3.04	0.0015	NA			1.73	1.13–2.67	0.012

CI: confidence interval; ICU: intensive care unit; Inf: Infinite value; NA: not applicable; OR: odds ratio.

^a^ The countries included are Belgium, Cyprus, Czechia, France, Greece, Ireland, the Netherlands, Sweden.

^b^ Severe outcome included ICU admission or death.

^c^ Number of cases included if differing from 1,176 cases: sex (n = 1,174); previous viral infections (n = 280); underlying conditions (n = 244); M type (n = 689).

We included 1,174 cases in the first multivariable analysis; two cases were excluded due to unknown sex. The age group 0–2 years was used as the reference age group for both models given it was the largest group (n = 320) with 45% of all ICU admissions. Males had a significantly lower likelihood of a severe outcome than females (OR = 0.57; 95% CI: 0.43–0.76; p < 0.001). Cases aged 6–10 years were less likely to have a severe outcome than the 0–2-year-olds (OR = 0.62; 95% CI: 0.42–0.92; p = 0.018). Severe outcome was more likely for cases with a lower respiratory tract diagnosis (OR = 4.14; 95% CI: 2.70–6.44; p < 0.001) and for those with a diagnosis of sepsis only (OR = 1.73; 95% CI: 1.11–2.73; p = 0.002).

In the severity analysis of 689 cases with typed isolates, severe outcome was more likely with *emm*1 compared with any other *emm* type (OR = 1.73; 95% CI: 1.13–2.67; p = 0.012). In this analysis, age and sepsis only were not associated with severe outcome. Cases with skin and soft tissue infections were less likely to have a severe outcome (OR = 0.40; 95% CI: 0.21–0.75; p = 0.005).

In an additional bivariate analysis of factors associated with ICU admission (regardless of death status: n = 280), a significant association with previous viral illness was seen (OR = 2.81; 95% CI: 1.16–7.88; p = 0.019), Supplementary Table S2. The same analysis was performed for factors associated with only death, but no significant associations were identified, as presented in Supplementary Table S3.

Previous viral illness and underlying conditions were tested as additional variables in the case-case severity analysis but showed no statistically significant differences between cases admitted to a ward and severe outcome (p = 0.664 and 0.756, respectively). This result is in line with the bivariate analysis for severe outcome for both these variables (p = 0.163 and 0.287, respectively, Table 4). In addition, unknown status for previous viral infection and underlying conditions was not associated with severe outcome.

## Discussion

In this collaborative multi-country study, we assessed the availability of iGAS surveillance data across EU/EEA countries and analysed pooled data on iGAS cases from several countries in Europe. Sharing of clinical information during the surge improved the detection of cases and risk communication on an emerging public health risk [18]. The recently established EUHTF was an effective mechanism for coordinating resources and facilitating operational research. Coordination was challenging partly due to the heterogeneous approaches to iGAS surveillance, as observed in our questionnaire results. Where iGAS surveillance is conducted, we found that methods for case finding varied, as did case definitions. The case definition used for this study was thus intentionally flexible, allowing for inclusion of cases from both laboratory-based and clinician-based case-finding.

The pooled data from the 2022/23 iGAS season showed sex and age to be associated with iGAS disease outcome severity. Males with iGAS disease had lower odds of ICU admission or death. Interestingly, in prior studies males were more frequently affected by iGAS disease than females [19], as observed in this study. However, to our knowledge, there are no prior studies on degrees of iGAS disease severity and sex.

Our findings are consistent with existing evidence that young children are at greatest risk for severe iGAS disease [8]. Cases aged 0–2 years accounted for 45% of ICU admissions and 37% of deaths. The case-case severity analysis showed lower odds of severe outcome for cases aged 6–10 years. We chose to include three birth years in our youngest age group, as these children aged ≤ 2 years likely shared a lack of prior exposure to GAS at the onset of the 2022/23 surge. Non-pharmaceutical interventions adopted during the COVID-19 pandemic contributed to reduced circulation of respiratory pathogens other than SARS-CoV-2. Subsequent surges in severe iGAS and other infectious diseases have been attributed, in part, to the accumulated lack of pathogen-specific immunity to protect against severe infection among those born during the pandemic. Additionally, older children might have experienced waning immunity over time with reduced exposures [20].

Patients with lower respiratory tract diagnoses had higher odds for severe outcomes, while patients with upper respiratory tract diagnoses and musculoskeletal diagnoses had lower odds of severe outcome. Upper respiratory tract infections and musculoskeletal infections are typically localised and rarely affect vital organs, whereas young children with signs of lower respiratory tract infections can deteriorate quickly with hypoxia. Public health messages to caregivers and clinicians during iGAS surges should therefore emphasise early recognition of lower respiratory tract infection.

Skin and soft tissue infections were associated with severe outcome only in the severity analysis for cases with typed isolates, when only cases with *emm* typing were considered. This discrepancy between the two models could be due to *emm* typing being more commonly done for isolates from patients presenting with severe skin and soft tissue infections, to identify strains associated with necrotising fasciitis [21]. For infection sites other than skin and soft tissue, *emm* typing might not be considered necessary for clinical purposes. Our surveillance questionnaire showed that some countries considered typing isolates from all iGAS cases useful, regardless of infection site, to understand the strains causing invasive disease. Typing of non-invasive isolates can be helpful to understand which strains are circulating, if a feasible sampling strategy can be implemented. Scarlet fever diagnoses are used as a proxy for iGAS surveillance in some countries, however further studies to understand the associations between scarlet fever incidence and iGAS incidence are needed.

Among countries reporting data on iGAS genotyping, *emm*1 was the dominant type, followed by *emm*12. These findings are consistent with findings from other European countries that did not contribute to the collection of case data, such as Denmark, Iceland and Portugal [22,23]. In addition, we found that infection with the *emm*1 strain was associated with severe outcome (p = 0.012). As shifts in the dominance of *emm* types may indicate considerable changes in GAS, monitoring of circulating GAS clones is important to understand when infections may spread more easily or be more severe. Identification of new dominant *emm* types could signal potentially altered transmission or virulence. Monitoring antimicrobial resistance in circulating GAS is also needed. Macrolide-resistant group A streptococci were recently included in the World Health Organization’s (WHO) priority list of antibiotic-resistant bacteria, classified as medium priority, further underscoring the need to enhance surveillance for GAS and address its impact [24]. Where resources are strained, representative sampling for typing and antimicrobial susceptibility testing should be considered to ensure cost-effectiveness while still providing valuable data.

Cases with viral illness had a higher likelihood of ICU admission (p = 0.019). Several studies show that respiratory viruses and varicella zoster infections increase the risk of iGAS [7,8,25,26]. Further evidence is needed to understand the dynamics that might cause iGAS cases to be more severe with coinfection or prior infection with certain viruses. As indicated by our surveillance questionnaire results, however, national public health institutes do not always have timely access to clinical laboratory test results for relevant viruses including influenza virus, varicella zoster virus and RSV. As public health disease surveillance systems evolve, integration of clinical data with disease surveillance will be important for studying risk factors associated with severe outcomes. Rapid sharing of clinical data to public health institutes can aid in understanding what contributes to severe illness and how to prevent it.

The significant association between previous viral infections and ICU admission in our study highlights the potential of childhood vaccination as a preventive measure to reduce ICU admissions and deaths associated with GAS. Robust vaccination strategies, particularly those targeting children and other vulnerable populations, not only reduce the burden of primary infections, but they can also lower the risk of secondary bacterial infections such as those caused by iGAS. In Australia and the UK, influenza vaccination in children is thought to be associated with decreased iGAS case numbers [7,27], and in Canada, vaccination programmes against varicella zoster have reduced varicella-associated iGAS [28]. In the United States (US), a decrease in paediatric varicella-related iGAS hospitalisations was also observed in 2004, coinciding with the use of the varicella vaccine [26]. Further investigations on the associations between childhood vaccinations and iGAS incidence can elucidate the impact of these vaccines on the morbidity and mortality of iGAS. As respiratory viral infections have been associated with iGAS infections, existing public health programmes for monitoring and controlling seasonal respiratory pathogens should consider integration of iGAS in their surveillance systems and prevention efforts. As with any pathogen transmitted via respiratory droplets, prevention and control measures and public health messages during respiratory virus season would apply: respiratory etiquette, hand hygiene, staying home when sick and early recognition of pneumonia in young children.

Strengthened surveillance with coordinated studies can improve our understanding of transmission dynamics and risk factors to generate targeted public health interventions for iGAS. For example, further studies can help tailor public messaging about symptom recognition or vaccination against respiratory viruses; surveillance can also determine optimal timing of public awareness and prevention messages. Operational research can also inform resource allocation for outbreak control such as strategies for antibiotic prophylaxis.

While our data were too sparse to study risk factors related to contact of an iGAS case in the same household, evidence from Australia, the UK and Canada [7,29,30] identifies this as a risk factor and supports antibiotic prophylaxis recommendations. Following the 2022/23 surge, some EU/EEA countries adopted antibiotic prophylaxis recommendations or adapted existing ones. Since the beginning of 2023, the Netherlands recommended prophylaxis for all household contacts of an iGAS case. Greece concurrently implemented a prophylaxis recommendation for all household contacts belonging to high-risk groups. Belgium had an existing recommendation for iGAS high-risk contacts, France recently updated an existing recommendation similarly [31], and Ireland recently included this in their national guidelines [32].

The main limitations of our study were data availability and data completeness, due to both a limited number of countries conducting iGAS surveillance, and difficulties in obtaining necessary clinical data. The need for medical chart reviews in many countries was indicated in responses to our surveillance questionnaire. The completeness of case reporting likely varies across countries due to variations in automated versus manual case-finding methods. Strengthened iGAS surveillance systems could facilitate larger multi-country iGAS studies in the future with more statistical power and clinical data to elucidate the clinical risk factors for severe disease and death. There is growing consensus that longer-term surveillance strategies for iGAS should be deployed in a coordinated manner across the EU/EEA countries [33]. A harmonised case definition for iGAS across multiple countries would facilitate the comparison of cases and the pooling of data across countries.

Case-case study design is not as robust as case-control analysis for assessing risk factors. However, this method was the best option to assess factors associated with severity among iGAS cases. Misclassification of outcomes was possible if ICU admission and death data were missing. Additionally, we acknowledge that some cases may have been missed if they died before diagnosis or if antibiotic treatment before testing led to culture-negative results; recognition of such cases varies across countries. Data on *emm*1 subtypes detected by WGS and other means during the 2022/23 season such as M1_global_, M1_UK_ and M1_DK_ [22,23] were not included in the study.

## Conclusion

We observed that countries strengthened their iGAS surveillance in response to the surge observed in the 2022/23 season by adopting new or supplementary retrospective and/or prospective monitoring methods. Integration of iGAS surveillance, prevention and control with public health programmes for other seasonal respiratory pathogens could be considered, as respiratory viral infections have been associated with iGAS infections and prevention and control measures would align. During respiratory virus season and when iGAS upsurges are detected, reminders to clinicians and the public about age-appropriate vaccinations and early recognition of sepsis and lower respiratory tract infection, particularly among children, could help prevent severe outcomes associated with iGAS. Strategies to determine trends in circulating *emm* types and macrolide resistance are warranted as potential early warning mechanisms for possible shifts in transmission patterns, virulence or resistance. Further studies on the protective effects of vaccines (e.g. influenza, varicella zoster) against iGAS could strengthen existing evidence for routine vaccinations. Recognising that EU/EEA countries will continue to differ in their capacities to detect, report and respond to iGAS outbreaks, collaborative frameworks – such as the one deployed for this study – can enhance cross-border surveillance, preparedness and response during public health crises.

## Data Availability

Case-based data could not be shared as they are qualified as personal data related to health and therefore might indirectly lead to the reidentification of the individual to which the data refer.

## Supplementary Data

274000 Supplementary Material
