# Supplementary material for: Multi-country surveillance of paediatric invasive group A Streptococcus infection, European Union/European Economic Area countries, 2022/23 season
Source: Euro Surveill. 2025 Oct 23;30(42):2500079. doi: 10.2807/1560-7917.ES.2025.30.42.2500079 (PMC12555117; doi:10.2807/1560-7917.ES.2025.30.42.2500079)
Supplement: Supplementary Material [file 25-00079_LEUNG_Supplement.pdf]

This supplementary material is hosted by Eurosurveillance as supporting information alongside the article "Multi-country surveillance of paediatric invasive group A *Streptococcus* infection, European Union/European Economic Area countries, 2022/23 season", on behalf of the authors, who remain responsible for the accuracy and appropriateness of the content. The same standards for ethics, copyright, attributions and permissions as for the article apply. Supplements are not edited by Eurosurveillance and the journal is not responsible for the maintenance of any links or email addresses provided therein.

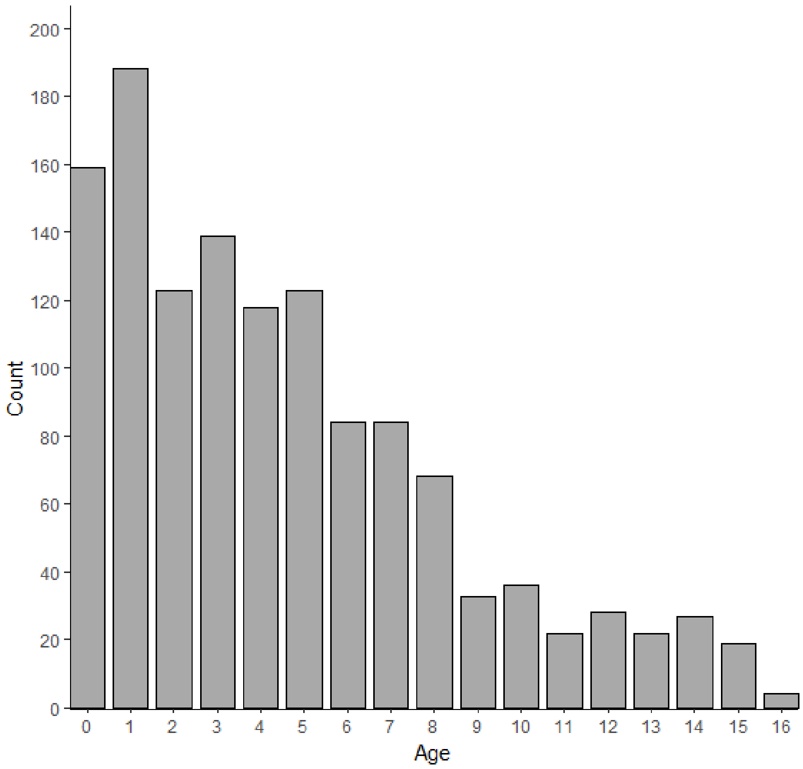

Figure S1 – Age distribution among invasive group A Streptococcus cases reported by participating countries (n=1277), the European Union/European Economic Area countries, 2022/23 season.

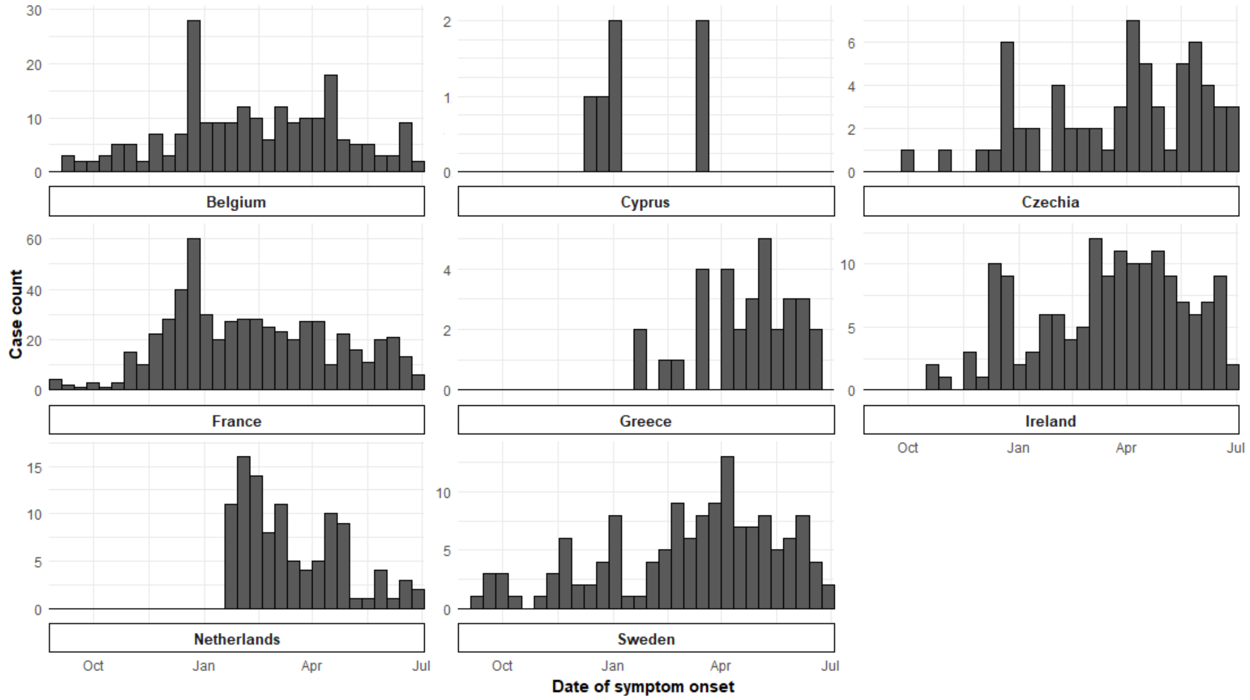

Figure S2 – Epidemic curves of the distribution of paediatric cases with invasive group A Streptococcus infections per participating country, by week of symptom onset, the European Union/European Economic Area countries, 1 September 2022–30 June 2023 (n=1,277).

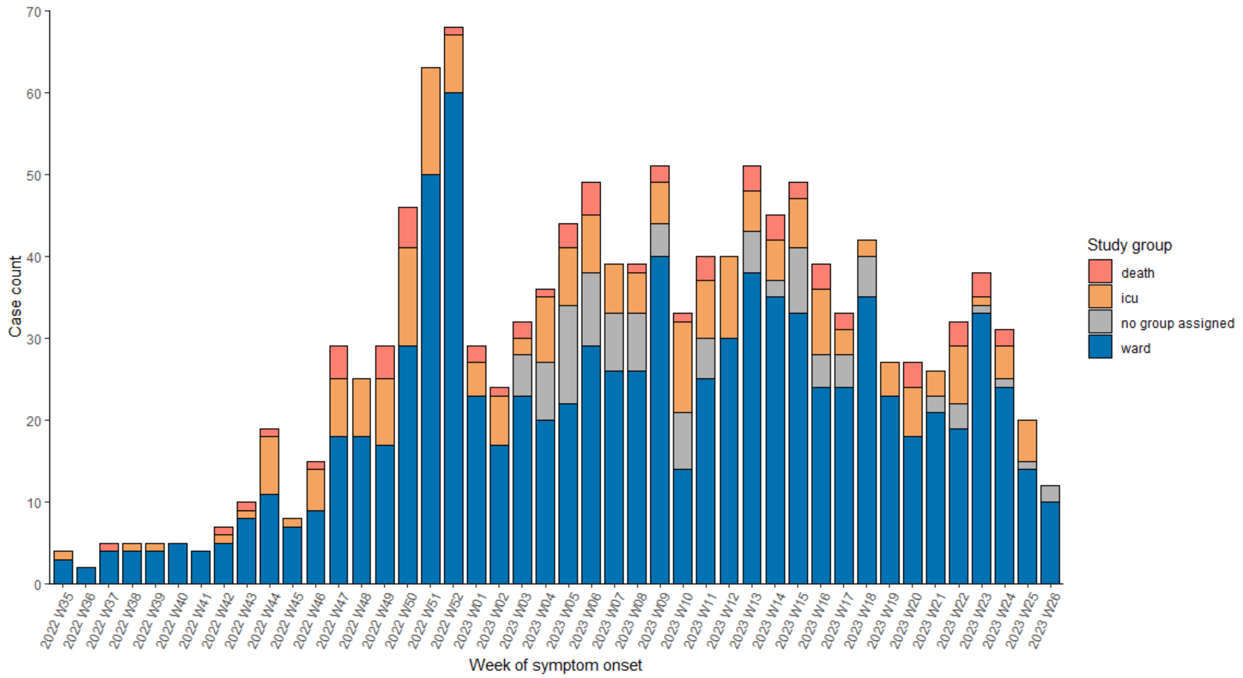

Figure S3 – Epidemic curve of the distribution of paediatric cases with invasive group A Streptococcus infections, by week of symptom onset, coloured by study group, the European Union/European Economic Area countries, 1 September 2022–30 June 2023 (n=1,277)

Table S1 – Diagnosis groups used in this study and the conditions included within them, participating countries, the European Union/European Economic Area countries, 2022/23 season. These groups were not mutually exclusive, one case could belong to more than one group depending on the reported diagnoses.

**Group**	**Diagnoses**
All sepsis diagnoses	Bacteraemia without focus<br>Septic shock,<br>STSS
Lower respiratory diagnoses	Pneumonia<br>Empyema
Upper respiratory diagnoses	Otitis media
Skin and soft tissue infections	Abscess<br>Cellulitis with systemic presentation<br>Necrotizing fasciitis<br>Postoperative wound infection
Musculoskeletal infections	Septic arthritis<br>Osteomyelitis<br>Myositis
Cardiac infections	Endocarditis<br>Pericarditis
Other	Chorioamnionitis<br>Peritonitis<br>Meningitis

Table S2 – Bivariate analysis for ICU admission only and variables with low completeness rate among reported cases estimated by Ficher's exact test, EU/EEA participating countries, 2022/23 season.

**Variables**	**Bivariate analysis**		
	OR	95% CI	P-value
Underlying conditions (n=244)	1.54	0.58–3.75	0.354
Previous viral infection (n=280)	2.81	1.16–7.88	0.019

Table S3 – Bivariate analysis for death only and variables with low completeness rate among reported cases estimated by Ficher's exact test, EU/EEA participating countries., 2022/23 season.

Variables	Bivariate analysis		
	OR	95% CI	P-value
Underlying conditions (n=244)	0.68	0.05- 3.01	0.635
Previous viral infection (n=280)	0.68	0.35-1.25	0.546

Supplementary File 1: Survey of iGAS surveillance across EU/EEA countries
This survey was distributed via REDCap between 21 September and 3 October 2023 to assess coverage and methodologies of existing surveillance systems for GAS and iGAS infections.

Survey of iGAS surveillance across EU/EEA countries

This survey is for all EU/EEA countries.The objectives of this survey are to: - Understand methods and limitations of EU/EEA country surveillance for invasive Group A Streptococcus (iGAS) infections. - For countries participating in the ECDC iGAS Project, understand which populations are included and excluded in case reporting of paediatric cases.The working case definition for reporting case data for the ECDC iGAS study is below.For analyses, the criteria marked with an asterisk (*) may be further restricted due to data availability from particpating countries. This survey therefore asks about inclusion and exclusion criteria for the cases reported to the ECDC iGAS project.Additionally, this survey will evaluate the ability of existing iGAS surveillance systems to detect iGAS cases defined by the case criteria.Study case definition for paediatrics iGAS infection:An illness in a person aged 0-16 years old* characterised by the criteria below resulting in hospitalization or death in one of the participating countries* from 1 September 2022 to 30 June 2023*.

Case criteria:

- Isolation by culture or detection by molecular methods (such as PCR) of Group A Streptococcus from a normally sterile body site (blood, cerebral spinal fluid, pericardium, peritoneum, pleural fluid, endometrium, joint aspirate, bone, deep tissue or deep abscess at operation or post-mortem).OR

- Isolation by culture or detection by immunoassay (rapid streptococcal test) or molecular methods (such as PCR) of Group A Streptococcus from a non-sterile body site (e.g., throat, ear, sputum, skin and nails, vagina, penis, anus, open wounds) AND clinical presentation consistent with severe streptococcal infection (STSS, puerperal sepsis, necrotising fasciitis, myositis, pneumonia, septic arthritis, meningitis, peritonitis, osteomyelitis, cellulitis with systemic presentation).

Country

◯ Austria ◯ Belgium
◯ Bulgaria ◯ Croatia
◯ Cyprus ◯ Czechia
◯ Denmark ◯ Estonia
◯ Finland ◯ France ◯ Germany
◯ Greece ◯ Hungary
◯ Iceland ◯ Ireland ◯ Italy
◯ Latvia ◯ Liechtenstein
◯ Lithuania ◯ Luxembourg
◯ Malta ◯ Netherlands
◯ Norway ◯ Poland ◯ Portugal
◯ Romania ◯ Slovakia
◯ Slovenia ◯ Spain ◯ Sweden

Country contact ______
Email address of contact person ______

Section 1: Existing public health surveillance systems prior to 2022 for Group A Streptococcus (GAS) and invasive Group A Streptococcus (iGAS) infections

Please indicate if public health surveillance of GAS and/or iGAS infections were implemented at a national, regional, and/or sentinel level prior to 2022.

Implemented prior to 2022

	GAS	iGAS
National surveillance	______	______
Regional surveillance	______	______
Sentinel surveillance	______	______

Regional surveillance refers to collection of data at a regional level, covering a catchment population of one or more geographic regions.

Sentinel surveillance refers to collection of data from select institutions, but not covering entire geographic regions.

Section 2: 2022/2023 paediatric case finding using existing and/or new or supplementary surveillance methods This setion asks if any additional surveillance methods were established during 2022/2023, and how cases reported to the ECDC iGAS project were identified. If not participating in the ECDC iGAS project, please answer "No" to question 2.3.

Were new or supplementary prospective iGAS surveillance methods established during the 2022-2023 GAS season? If yes, please provide start date of prospective surveillance.

______ ______

Were new or supplementary retrospective iGAS surveillance methods established during the 2022-2023 GAS season? If yes, please provide start and end dates of retrospective surveillance

______ ______ - ______

Please indicate all methods used for reporting GAS and/or iGAS cases to public health authorities.

Laboratory reporting (positive lab finding triggers case reporting) ______
Clinician reporting (example: clinicians required to report iGAS cases) ______

Is [country_v2] participating in the ECDC iGAS project? ◯ Yes ◯ No

You have indicated:

- No GAS or iGAS surveillance prior to 2022 was indicated in Section 1.
- No establishment of prospective or retrospective iGAS surveillance during 2022/2023 in Section 2.
- Not participating in the ECDC iGAS project. If [country_v2] has any iGAS surveillance OR is participating in the ECDC iGAS Project, please edit your response(s) above to indicate case finding methods used.

If all of the above are true, this is the end of the survey.

Is there any other information you would like to share regarding GAS or iGAS in your country?

You have indicated:

- No GAS or iGAS surveillance prior to 2022 was indicated in Section 1.
- No establishment of prospective or retrospective iGAS surveillance during 2022/2023 in Section 2.
- Participating in the ECDC iGAS project. If [country_v2] is participating in the ECDC iGAS Project, please edit your response(s) above to indicate case finding methods used.

Were reported cases detected using existing public health surveillance systems for GAS (as indicated in Section 1 above)?

______

______

Were reported cases detected using existing public health surveillance systems or iGAS (as indicated in Section 1 above)?

______

______

If neither laboratory nor clinician reporting are used, how are cases found?

____

Which GAS laboratory findings are reported?

______ ______

What is the definition of iGAS used for clinician reporting?

____

Section 3: Limitations to case finding methods: alignment with ECDC study definition This section asks if inclusion criteria for the ECDC iGAS project are commpletely met by case finding and what more restrictive criteria are used (if any) for reported cases.

The following are inclusion criteria for ECDC's paediatric iGAS infection study:

- ages 0-16
- all hospitalisations for iGAS
- all deaths associated with iGAS
- country-wide for participating countries
- hospitalisation or death during 1 September 2022 to 30 June 2023
- GAS isolated from a normally sterile body site OR GAS isolated from a non-sterile body site and clinical presentation of severe streptococcal infection. 3.1 If there are limitations in the established surveillance methods that would lead to incomplete case finding according to the ECDC iGAS study definition of paediatric iGAS cases resulting in hospitalisation or death, please indicate the ECDC inclusion criteria that are not completely covered by the GAS/iGAS cases reported to public health authorities in your country.

______

Age criteria incomplete

What are the minimum and maximum ages (in years) included in reported cases?

Minimum age: ______ Maximum age: ______

Hospital criteria or date range incomplete

What were the start and end dates used for case finding of reported hospitalised cases?

Start date: ______ End date: ______

Are hospital admissions prior to [admissions_start] included (if the patient was still hospitalised on the start date for inclusion)?

______

Death criteria or date range incomplete

What were the start and end dates used for case finding of reported iGAS cases associated with death?

Start date: ______ End date: ______

Please briefly explain inclusion criteria for reporting to ECDC's iGAS project if they differ from the study case definition.

Section 4: Methods for collecting case data This section asks about ease of collecting "essential" case data points for iGAS cases and also detailed clinical data points.

Essential data points for the ECDC iGAS study are below and are underlined in the REDCap case report form for the project.

1.3 - Date of data extraction

1.4 - Admission to ward

1.5 - Admission to ICU

1.6 - Date of illness onset for Group A Streptococcus (GAS) infection.

1.7 - Age

1.8 - Sex

1.9 - Death

1.10 - Type(s) of invasive infection caused by Group A Streptococcus

5.1 - Date of collection for first sample that tested positive for Group A Steptococcus (GAS)

5.3 - Site(s) of positive GAS specimen(s) (including specifying the body site if 'other' is selected)

7.2 - Date of death

4.1 Are all "essential" data points reported to the public health agency for GAS/iGAS cases?

◯ Yes - all essential data are reported and no further investigation is required to submit essential data for the cases.
◯ No - not all essential data are reported. Further investigation is required to submit essential data.

4.1.1 Which data points are NOT reported and thus require further investigation/inquiry to obtain? (Please check all that are not reported to public health agency)

☐ Admission to ward (Y/N)
☐ Date of admission to ward
☐ Admission to ICU (Y/N)
☐ Date of admission to ICU
☐ Symptom onset date for GAS infection
☐ Age of patient (at hospitalisation and/or death)
☐ Sex of patient
☐ Death status of patient
☐ Date of death
☐ Type(s) of invasive infection caused by GAS
☐ Date of collection for first sample that tested positive for GAS
☐ Anatomic site(s) of positive GAS specimen(s)

4.1.2 While not all reported, can all "essential" data points be made available to the public health agency without the need for medical chart review?

If these data are not immediately available but can be made available upon further inquiry, please click "Yes".

If all data points cannot be made available without a medical chart review, please check "No".

◯ Yes - all essential data can be available to the public health agency if requested without needing medical chart review.
◯ No - not all essential data is available without medical chart review.

4.1.3 Which data points are NOT available unless the medical chart is reviewed?

Note: if a data point is checked here, it should also be checked in the list of "not reported data points" above. (Please check all data points that are not available without chart review)

☐ Admission to ward (Y/N)
☐ Date of admission to ward
☐ Admission to ICU (Y/N)
☐ Date of admission to ICU
☐ Symptom onset date for GAS infection
☐ Age of patient (at hospitalisation and/or death)
☐ Sex of patient
☐ Death status of patient
☐ Date of death
☐ Type(s) of invasive infection caused by GAS
☐ Date of collection for first sample that tested positive for GAS
☐ Anatomic site(s) of positive GAS specimen(s)

Molecular typing

4.2 Was emm typing or whole genome sequencing routinely done for GAS isolates of reported iGAS cases during 2022/2023?

emm typing ______
Whole genome sequencing ______

Additional clinical data such as healthcare visits prior to admission, viral co-infections and medical predisposition (including vaccination status), and antibiotics given are sometimes available to public health agencies.

4.3 Please indicate which types of data are available to the public health agency.

☐ outpatient visit dates
☐ outpatient visit diagnoses
☐ diagnoses at hospital admission or discharge
☐ clinical laboratory test results for viruses including influenza, varicella, and RSV
☐ all medical diagnoses
☐ vaccination records
☐ antibiotics administered during and after hospitalisation
☐ none of the above

Thank you for completing this survey of iGAS surveillance methods in EU/EEA countries.

Is there any other information you would like to share regarding case finding or collection of case data for iGAS in your country?

Supplementary File 2: iGAS case report form
This case report form in REDCap was used to collect data from national contact points regarding each case fulfilling the study case definition. This form was also converted to Microsoft Excel format upon request from national contact points.

iGAS hospitalised case report form

Record ID

____
(This ID is automatically generated and can be used for your internal tracking purposes)

This ECDC Invasive Group A Streptococcus (iGAS) case report form is for a retrospective study on iGAS infections during the 2022-2023 season with the following objectives:

Describe iGAS cases that occurred in participating European countries among the paediatric population during the 2022-2023 season.

Retrospectively assess available data on paediatric iGAS cases in collaborating countries to better understand the risk factors for progression to severe illness and death, particularly the role of coinfections, with the goal of developing targeted prevention messages for future GAS seasons.

Evaluate the availability of data pertaining to GAS and iGAS cases across the EU/EEA and consider potential long-term recommendations for surveillance.

Please complete one form (record) for each case, as determined by the case definition below.

Essential variables are underlined and include data regarding:

- Demographics, admission date(s), onset date, and type of infection(s) (in Section 1)
- First GAS collection date and site(s) where GAS was detected (in Section 5)
- Date of death (if died, in Section 7) Records may be saved for completion at a later time.

When a record is ready to be incorporated into the dataset for analyses, please indicate "Complete" at the bottom of the form and save.

Questions about this data collection form can be emailed to EUHTF@ecdc.europa.eu

Powered by REDCap

The working case definition for this study is:

An illness in a person aged 0-16 years old* characterised by the criteria below resulting in hospitalization or death in one of the participating countries* from 1 September 2022 to 30 June 2023*.

Case criteria:

- Isolation by culture or detection by molecular methods (such as PCR) of Group A Streptococcus from a normally sterile body site (blood, cerebral spinal fluid, pericardium, peritoneum, pleural fluid, endometrium, joint aspirate, bone, deep tissue or deep abscess at operation or post-mortem).

OR

- Isolation by culture or detection by immunoassay (rapid streptococcal test) or molecular methods (such as PCR) of Group A Streptococcus from a non-sterile body site (e.g., throat, ear, sputum, skin and nails, vagina, penis, anus, open wounds) AND clinical presentation consistent with severe streptococcal infection (STSS, puerperal sepsis, necrotising fasciitis, myositis, pneumonia, septic arthritis, meningitis, peritonitis, osteomyelitis, cellulitis with systemic presentation). *For analyses, age, geographical, and time-based criteria may be further restricted due to data availability. Please report all cases with available data that correspond to this case definition. A separate survey will be conducted to understand each country's reporting limitations to inform analysis.

Section 1: Patient demographics and dates

1.1 Country of residence

Optional.

◯ Afghanistan
◯ Albania
◯ Algeria
◯ Andorra
◯ Angola
◯ Antigua and Barbuda
◯ Argentina
◯ Armenia
◯ Australia
◯ Austria
◯ Azerbaijan
◯ Bahamas
◯ Bahrain
◯ Bangladesh
◯ Barbados
◯ Belarus
◯ Belgium
◯ Belize
◯ Benin
◯ Bhutan
◯ Bolivia
◯ Bosnia and Herzegovina
◯ Botswana
◯ Brazil
◯ Brunei
◯ Bulgaria
◯ Burkina Faso
◯ Burundi
◯ Cabo Verde
◯ Cambodia
◯ Cameroon
◯ Canada
◯ Central African Republic
◯ Chad
◯ Chile
◯ China
◯ Colombia
◯ Comoros
◯ Congo, Democratic Republic of the
◯ Costa Rica
◯ Cote d'Ivoire
◯ Croatia
◯ Cuba
◯ Cyprus
◯ Czechia
◯ Denmark
◯ Djibouti
◯ Dominica
◯ Dominican Republic
◯ Ecuador
◯ Egypt
◯ El Salvador
◯ Equatorial Guinea
◯ Eritrea
◯ Estonia
◯ Eswatini
◯ Ethiopia
◯ Fiji
◯ Finland
◯ France
◯ Gabon
◯ Gambia
◯ Georgia
◯ Germany
◯ Ghana
◯ Greece
◯ Grenada
◯ Guatemala
◯ Guinea

Powered by REDCap

◯ Guinea-Bissau
◯ Guyana
◯ Haiti
◯ Honduras
◯ Hungary
◯ Iceland
◯ India
◯ Indonesia
◯ Iran
◯ Iraq
◯ Ireland
◯ Israel
◯ Italy
◯ Jamaica
◯ Japan
◯ Jordan
◯ Kazakhstan
◯ Kenya
◯ Kiribati
◯ Kosovo
◯ Kuwait
◯ Kyrgyzstan
◯ Laos
◯ Latvia
◯ Lebanon
◯ Lesotho
◯ Liberia
◯ Libya
◯ Liechtenstein
◯ Lithuania
◯ Luxembourg
◯ Madagascar
◯ Malawi
◯ Malaysia
◯ Maldives
◯ Mali
◯ Malta
◯ Marshall Islands
◯ Mauritania
◯ Mauritius
◯ Mexico
◯ Micronesia
◯ Moldova
◯ Monaco
◯ Mongolia
◯ Montenegro
◯ Morocco
◯ Mozambique
◯ Myanmar
◯ Namibia
◯ Nauru
◯ Nepal
◯ Netherlands
◯ New Zealand
◯ Nicaragua
◯ Niger
◯ Nigeria
◯ North Korea
◯ North Macedonia
◯ Norway
◯ Oman
◯ Pakistan
◯ Palau
◯ Palestine
◯ Panama
◯ Papua New Guinea
◯ Paraguay
◯ Peru
◯ Philippines
◯ Poland
◯ Portugal

◯ Qatar
◯ Romania
◯ Russia
◯ Rwanda
◯ Saint Kitts and Nevis
◯ Saint Lucia
◯ Saint Vincent and the Grenadines
◯ Samoa
◯ San Marino
◯ Sao Tome and Principe
◯ Saudi Arabia
◯ Senegal
◯ Serbia
◯ Seychelles
◯ Sierra Leone
◯ Singapore
◯ Slovakia
◯ Slovenia
◯ Solomon Islands
◯ Somalia
◯ South Africa
◯ South Korea
◯ South Sudan
◯ Spain
◯ Sri Lanka
◯ Sudan
◯ Suriname
◯ Sweden
◯ Switzerland
◯ Syria
◯ Taiwan
◯ Tajikistan
◯ Tanzania
◯ Thailand
◯ Timor-Leste
◯ Togo
◯ Tonga
◯ Trinidad and Tobago
◯ Tunisia
◯ Turkey
◯ Turkmenistan
◯ Tuvalu
◯ Uganda
◯ Ukraine
◯ United Arab Emirates (UAE)
◯ United Kingdom (UK)
◯ United States of America (USA)
◯ Uruguay
◯ Uzbekistan
◯ Vanuatu
◯ Vatican
◯ Venezuela
◯ Vietnam
◯ Yemen
◯ Zambia
◯ Zimbabwe

1.2 Hospital where patient was treated

Optional. For evaluating number of hospitals included and distribution of cases among hospitals.

____
(Please assign (non-identifiable) numerical codes for hospitals)

1.3 Date of data extraction

Date when data were retrieved from medical record or other source.

____

1.4 Was the patient admitted to a hospital ward (non-ICU) for iGAS? ______
If yes, please provide date of first admission to a hospital ward on/after date of iGAS onset. ______

1.5 Was the patient admitted to an intensive care unit (ICU) or iGAS? ______
If yes, please provide date of first admission to an ICU on/after date of iGAS onset. ______

1.6 Date of illness onset for Group A Streptococcus (GAS) infection.

If symptom onset date not available, provide date of earliest record of GAS infection (first healthcare visit date or positive test result, whichever is first)

____
(Date should be between 01-08-2022 and 01-07-2023)

____

____

1.7 Age of patient on the date of illness onset ([date_sympt_onset]) Age ______

______

If less than one year old, please indicate age in days or months.

Infant's gestational age and weight at birth ______ weeks
(if available) ______ days
______ grams

1.8 Sex ◯ Male ◯ Female

1.9 Has the patient died? ◯ Yes ◯ No ◯ Unknown

As of date of data abstraction [date_medical_record_review]

1.10 Type(s) of invasive infection caused by Group A Streptococcus

Please mark ALL diagnoses from date of onset to time of medical record review

- ☐ Abscess (deep, do not include superficial skin abscess)
- ☐ Bacteremia without Focus
- ☐ Cellulitis without systemic presentation
- ☐ Cellulitis with systemic presentation
- ☐ Chorioamnionitis
- ☐ Endocarditis
- ☐ Epiglottitis
- ☐ Endometritis
- ☐ Empyema
- ☐ Hemolytic uremic syndrome (HUS)
- ☐ Meningitis
- ☐ Myositis
- ☐ Necrotizing fasciitis
- ☐ Osteomyelitis
- ☐ Otitis media
- ☐ Peritonitis
- ☐ Pericarditis
- ☐ Pneumonia
- ☐ Postoperative wound infection
- ☐ Puerperal sepsis
- ☐ Septic abortion
- ☐ Septic arthritis
- ☐ Septic shock
- ☐ STSS

2.1 Previous healthcare visits for this episode of GAS infection.

If medical encounters prior to hospital admission are noted in the medical record, please indicate the date and location of the first medical visit for this illness.

If a second medical encouter occurred prior to admission, please indicate that as well.

______

______ ______
______ ______

Section 3: Clinical presentation at hospital admission

3.1 Please indicate symptoms documented in the patient's chart at presentation prior to admission.

Fever ______
Coryza ______
Cough ______
Vomiting ______
Diarrhoea ______
Cellulitis or soft tissue infection ______
Respiratory distress ______
Poor feeding ______
Rash ______
Pharyngitis ______
Ear pain ______
Bone or joint pain ______

3.2 Please indicate examination findings documented in the patient's chart at admission.

Temperature at presentation (degrees Celcius) ______
Presence of lymphadenopathy ______
Skin barrier breach (e.g. trauma, wound, bite) ______
Sepsis at presentation ______
Septic shock at presentation ______

Powered by REDCap

Site of cellulitis/soft tissue infection

____

Section 4: Viral co-infections and medical predisposition

4.1 Please indicate any respiratory viral infection diagnoses during the following time period:

from: 30 days prior to iGAS illness onset ([onset_day_minus30])

to: 30 days after iGAS illness onset ([onset_day_plus30])

Diagnosis can be on the basis of a positive test or clinical judgement. Diagnostic codes for may be used to determine coinfections.

If a negative test was obtained, please choose "negative". To avoid diagnostic bias, cases with "negative test" data will not be considered uninfected cases; these data can be used to inform testing patterns.

If there is no record of testing or diagnosis, choose "unknown".

Please provide a date of diagnosis if known (positive test date or date of clinical diagnosis, whichever was first).

Virus	Diagnosis during time period above	Date of diagnosis
Varicella	______	______
SARS-CoV-2	______	______
Respiratory syncytial virus	______	______
Influenza	______	______
Other positive viral test	______	______

4.2 Please indicate vaccination/immune status for the following viruses on date of iGAS illness onset ([date_sympt_onset], response to question 1.6

Varicella vaccinated/immune?	______
COVID-19 vaccinated?	______
Respiratory syncytial virus prophylaxis?	______
Influenza vaccinated for 2022-2023 season?	______
Measles immune?	______

4.3 Did the patient have any of these underlying medical conditions?

Check all that apply.

If none, please check "None of the above".

If unknown, leave blank.

- ☐ Asthma
- ☐ Other chronic lung disease (other than asthma)
- ☐ Diabetes
- ☐ Other immunocompromising condition
- ☐ None of the above

Please specify the immunocompromising condition

____

Was the patient pregnant within 3 months of the illness onset date?

◯ Yes ◯ No ◯ Unknown

4.4 Did this patient have any other illness in the 30 days prior to illness onset date (starting [onset_day_minus30])?

◯ Yes ◯ No ◯ Unknown

Please specify diagnosis for prior illness.

____

Powered by REDCap

4.5 Did another household member have a documented GAS infection in the 30 days preceding date of illness onset (since [onset_day_minus30])? ◯ Yes ◯ No ◯ Unknown

Section 5: Group A Streptococcus microbiological testing

5.1 Date of collection for first sample that tested positive for Group A Steptococcus (GAS)

____
(Must be on/after onset date and on/before data extraction date.)

5.2 Which tests were conducted for detection of Group A Streptococcus?

If multiple tests of the same type were conducted, please choose "positive" if any were positive, and "negative" if all were negative".

If test type was not used, please choose "unknown".

Bacterial culture ______
Molecular test (PCR) ______
Antigen test ______
Other method (describe) ______

5.3 Site(s) of positive GAS specimen(s)

Please indicate all body part(s) from which GAS was detected.

- ☐ Blood
- ☐ Cerebrospinal fluid (CSF)
- ☐ Pharyngeal swab
- ☐ Pleural fluid
- ☐ Skin/soft tissue
- ☐ Bone/joint
- ☐ Peritoneal/ascites fluid
- ☐ Other

Please specify other body site

____

5.4 emm type (if available)

____

5.5 Is the isolate/specimen available? ◯ Yes ◯ No ◯ Unknown

This patient has died (response to question 1.9). Was GAS detected post-mortem? ◯ Yes ◯ No ◯ Unknown

Date of isolation of a GAS bacterium

If multiple isolates, provide first date of isolation

____
(Must be after first date of collection and before data extraction date.)

Did the isolate undergo antibiotic susceptibility testing? ◯ Yes ◯ No ◯ Unknown

Please indicate the antibiotics to which the isolate was not susceptible.

If susceptible to all antibiotics tested, please check the last box.

If susceptibility testing done but test results not available, please check "unknown".

- ☐ Amoxcicillin
- ☐ Azithromycin
- ☐ Clindamycin
- ☐ Erythromycin
- ☐ Levofloxacin
- ☐ Moxifloxacin
- ☐ Penicillin G
- ☐ Tetracycline
- ☐ Trimethoprim-sulfamethoxazole
- ☐ Vancomycin
- ☐ Other
- ☐ Unknown
- ☐ Susceptible to all antibiotics tested

Please specify other antibiotic for which the isolate was not susceptible. ______________________________

Section 6: Course of illness and treatment

6.1 Cardiac arrest occurred during course of iGAS infection. ◯ Yes ◯ No ◯ Unknown

6.2 Respiratory arrest occurred during course of iGAS infection. ◯ Yes ◯ No ◯ Unknown

6.3 Mechanical ventilation administered? ◯ Yes ◯ No ◯ Unknown

6.4 IVIG therapy administered? ◯ Yes ◯ No ◯ Unknown

6.5 Renal Replacement Therapy administered? ◯ Yes ◯ No ◯ Unknown

6.6 Please list the antibiotics administered, along with the start and end dates of each course of therapy.

Antibiotic name Start date Stop date

______ ______ ______

______ ______ ______

______ ______ ______

If more than 3 antibiotics have been given, please write details in the comments box at the bottom of this form.

Section 7: Outcomes Please indicate if the patient has died (section 1, question 1.9) before completing this section.

7.1 This patient died (response to question 1.9).

When/Where did the patient die?

- ◯ Before reaching the hospital for iGAS treatment (outside hospital)
- ◯ During hospital admission for iGAS treatment (in hospital)
- ◯ After discharge from hospital for iGAS treatment (outside hospital)
- ◯ Unknown

7.2 Please specify the date of death. ______________________________
(Must be on/after onset date and on/before data extraction date.)

Powered by REDCap

7.1 Has the patient been discharged from the hospital as of data abstraction date ([date_medical_record_review])?<br><br>If still hospitalised, choose 'No'.<br><br>If no record of discharge or continued admission, choose "Unknown".	◯ Yes ◯ No ◯ Unknown
Date of discharge from the hospital<br><br>If multiple hospital admissions, provide date of last discharge associated with iGAS treatment.	________________________________<br>(Must be on/after onset date and on/before data extraction date.)
Was the patient discharged with outpatient parenteral antimicrobial therapy (OPAT)?	◯ Yes ◯ No ◯ Unknown
Please specify the duration of OPAT.	________________________________
7.2 Did the patient recover without complications?	◯ Yes ◯ No ◯ Unknown
7.3 Is the patient experiencing long-term sequelae due to the iGAS infection?<br><br>Examples of long-term sequelae include reduced musculoskeletal, pulmonary, renal or other function.	◯ Yes ◯ No ◯ Unknown
Please briefly describe any documented long-term sequelae.	________________________________

End of case report form.

Please check entered data for accuracy, with special attention to essential (underlined) variables, before saving with "complete" status.

Additional information	________________________________<br>(Please write any additional information you would like to provide about this patient/case.)
